# Cracking the Sugar Code by Mass Spectrometry: An Invited Perspective in Honor of Dr. Catherine E. Costello, Recipient of the 2017 ASMS Distinguished Contribution Award

**DOI:** 10.1007/s13361-018-1912-3

**Published:** 2018-04-11

**Authors:** Ekaterina Mirgorodskaya, Niclas G. Karlsson, Carina Sihlbom, Göran Larson, Carol L. Nilsson

**Affiliations:** 1 Proteomics Core Facility, University of Gothenburg, Sahlgrenska Academy, Box 413, SE-405 30 Gothenburg, Sweden; 2 Department of Medical Biochemistry, University of Gothenburg, Sahlgrenska Academy, Box 440, SE-405 30 Gothenburg, Sweden; 3 Department of Clinical Chemistry and Transfusion Medicine, University of Gothenburg, Sahlgrenska Academy, Institute of Biomedicine, SE-413 45 Gothenburg, Sweden; 4 Department of Experimental Medical Science, Lund University, SE-223 62 Lund, Sweden

**Keywords:** Glycan mass spectrometry, History of mass spectrometry, Structural mass spectrometry, Review

## Abstract

The structural study of glycans and glycoconjugates is essential to assign their roles in homeostasis, health, and disease. Once dominated by nuclear magnetic resonance spectroscopy, mass spectrometric methods have become the preferred toolbox for the determination of glycan structures at high sensitivity. The patterns of such structures in different cellular states now allow us to interpret the sugar codes in health and disease, based on structure-function relationships. Dr. Catherine E. Costello was the 2017 recipient of the American Society for Mass Spectrometry’s Distinguished Contribution Award. In this Perspective article, we describe her seminal work in a historical and geographical context and review the impact of her research accomplishments in the field.

8

**Figure:**
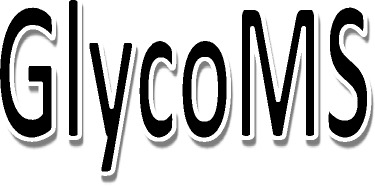
ᅟ Graphical abstract

ᅟ

Graphical abstract

## Introduction

Structural elucidation of glycans and glycoconjugates is still not a trivial pursuit, but structural aspects of glycobiology are essential to assign glycoforms’ involvement in homeostasis, health, and disease. Nuclear magnetic resonance spectroscopy was the first important structural method for glycoconjugate analysis. However, during the past 35 years, sensitive and specific mass spectrometric (MS) tools have been developed for the determination of glycan structures. The determination of temporal patterns of glycostructures in different cellular states now enables the interpretation of “sugar codes” in health and disease. Dr. Catherine E. Costello, a pioneering scientist in the field of biological mass spectrometry, was the 2017 recipient of the American Society for Mass Spectrometry’s Distinguished Contribution Award. In this Perspective article, we describe her achievements against the background of major developments in the field and her successful international collaborations.

## The Early Days of Biological Mass Spectrometry: A Dynamic Global Environment

At the time that Dr. Costello entered the group of Klaus Biemann at Massachusetts Institute of Technology, Boston had already become the major hub of the mass spectrometry field: fundamentals and applications. Logan Airport became a frequent destination for global MS enthusiasts, who visited the Biemann lab and participated in summer Gordon Conferences in the New England area. In the early days, the modalities for molecular desorption/ionization were limited. Biemann was a close friend of Einar Stenhagen (Gothenburg University (GU), Sweden); together, they were considered the founders of organic mass spectrometry. In his presentations, Biemann often mentioned the first successful peptide sequence by MS, performed in Stenhagen’s lab by his student, Carl-Ove Andersson [1]. Stenhagen’s laboratory was located at the Department of Medical Biochemistry (GU). His partner in life and mass spectrometry, Professor Stina Stenhagen, was a skillful MS operator and experimentalist. In 1964, Einar Stenhagen filed a patent describing the first successful hyphenation of gas chromatography (GC) to MS (the Becker jet separator). A Stenhagen protégé, Ragnar Ryhage of Karolinska Institute, purchased the first commercial GC-MS (LKB 9000). The instrument was used to characterize prostaglandins, a project that was awarded the 1982 Nobel Prize in physiology or medicine to Sune Bergström, Bengt Samuelsson, and John Vane.

A now classic review paper was published by Einar Stenhagen in the *Journal of Lipid Research* in 1960 [2]. A young student of Stina Stenhagen, Karl-Anders (“Kacka”) Karlsson, was the first to identify human kidney-derived phytosphingosine by use of Stenhagen’s GC-MS, as a trimethylsilyl derivative [3]. Previously, he had published his single author report in *Nature* [4] on the separation of sphingosine bases of glycolipids by reversed phase paper chromatography. During a visit of Biemann to Gothenburg, he shared his discovery of amide reduction to amine by use of LiAlH_4_. This new knowledge inspired Karlsson to develop permethylation and permethylated-reduced glycolipids for MS analysis [5], later used by Sen-itiroh Hakomori in 1974 to characterize a hexasaccharide-containing human brain ganglioside: sequence, branching, and fatty acid and sphingosine composition [6]. Karlsson became a lifelong friend and frequent visitor of Biemann. He was an admirer of Biemann’s key contributions to NASA’s Viking 1 project, in which a mass spectrometer designed by Biemann landed on Mars in 1976. Biemann generously offered his own personal copy of the published report to Karlsson.

It was into this dynamic international environment that Dr. Costello entered and embarked her journey into biological mass spectrometry, when the field was very young. During the 1970s, Dr. Costello worked in the organic mass spectrometry field, emphasizing GC-MS for analysis of drugs in bodily fluids [7–9]. During this era, she began a longstanding interest in lipid chemistry, biochemistry, and analysis [10, 11]. At this time, GC-MS was the method of choice for biomolecular analysis; however, it was necessary to convert polar molecules to non-polar derivatives in order to increase their volatilities to the point that GC was feasible. GC-MS employs electron impact ionization, a technique that does not allow production of intact biomolecular ions, even after their derivatization. Field desorption was developed as a means of ionizing thermally labile compounds [12]. This technique involved coating the analyte on to an emitter consisting of a thin wire covered with field enhancing micro-needles. The needles concentrated an applied potential to create an ion current that could be sampled using a mass analyzer. Dr. Costello was a pioneer in applying field desorption to lipids [13–17] as well as organic and organometallic compounds [18].

The invention of fast atom bombardment [19] (also known as liquid secondary ion mass spectrometry) was significant; in that, it allowed for the first time routine analysis of underivatized biomolecules. To prepare samples, one added a microliter or so of glycerol (or other viscous hydrophilic molecule) to a biomolecule sample solution and then evaporated the water and/or organic solvent in vacuo. The sample, now concentrated into glycerol, was applied to a metal probe and placed in the MS vacuum source. A beam of atoms or ions was then used to sputter the glycerol-biomolecule solution into the gas phase for MS analysis. Dr. Costello was among the first to apply FAB-MS to peptide analysis [20–22]. It is important to recall that the principles behind collisional tandem MS methods for biomolecules in use today were developed using FAB ionization. Dr. Costello pioneered the application of FAB-tandem MS to lipids [17, 23–25]. The FAB technique was relatively more energetic than the soft ionization methods in use today, often necessitating permethylation to stabilize glycans and improve their ionization. Dr. Costello published a seminal article on tandem mass spectrometric analysis of permethylated glycans [26] and glycolipids [25] that defined the product ion nomenclature that is used worldwide.

Dr. Costello’s very first work was in toxicology assays by MS, but she soon initiated molecular studies using a high-energy CID-enabled four-sector instrument (JEOL). She began her foray into biological MS through the development of modes and methods tailored to advanced projects in carbohydrate molecular weight profiling, sequence, linkage, and branching data [27, 28] derived from scarce biological samples [23]. Later, the establishment of her independent research group at Boston University School of Medicine allowed her to expand her network of collaborators and cadre of international mentees.

## Enabling Technological Developments in Biological Mass Spectrometry

The introduction of soft ionization techniques, electrospray ionization (ESI), and matrix-assisted laser desorption/ionization (MALDI) had a major impact on the development of modern biological mass spectrometry. Their discovery was awarded the Nobel Prize in Chemistry in 2002. Due to the ability to produce large intact molecular ions, these techniques enabled the study of biomolecules that had previously been intractable to gas phase analysis. The main interest was directed toward ionization and detection of large molecules such as proteins; few researchers focused on glycoconjugates at that time, with Dr. Costello being a leader.

From the outset, it was clear that the success of MALDI analysis strongly depended on the selection of MALDI matrix and sample preparation technique. Dozens of potential matrix compounds were tested by different groups, and preferred matrices for protein and peptide analysis were quickly identified: α-cyano-4-hydroxycinnamic acid (CHCA) [29], 2,5-dihydroxybenzoic acid (DHB) [30], and 3,5-dimethoxy-4-hydroxycinnamic acid (sinapinic acid) [29]. Even after the successful application of MALDI to oligosaccharide analysis had been demonstrated [31], just a few groups focused on MALDI desorption methods for glycoconjugate analysis. Dr. Costello was among those who pioneered MALDI-MS analysis of glycoconjugates. Carbohydrates can be linked to different types of molecules such as lipids, proteins, and peptides, and the conditions for glycoconjugate analysis are affected by both moieties. HABA (2-(4-Hydroxyphenylazo)benzoic acid) was among the MALDI matrices investigated by Dr. Costello [32] and was found to be particularly useful in the analysis for larger glycoproteins. Since its discovery as a MALDI matrix, HABA has been found to be useful for analysis of various molecules. Recently, ionic liquid matrices based on HABA were shown to be suitable for analysis of polysulfated carbohydrates such as heparin and heparan sulfate, enabling detection of intact fully *O*- and *N*-sulfated species [33]. A recent comparison of MALDI wavelength dependence of a number of well-established matrices showed unique properties of HABA [34]. HABA has a broad absorption band in the solid state from 360 to 500 nm with optimal MALDI ion yields between about 380 and 440 nm. Among all investigated matrices, HABA was the only one resulting in successful MALDI using visible laser light. HABA was able to produce analyte ions above 500 nm; reduced fragmentation was reported with visible laser light.

With Dr. Costello’s extensive experience in the analysis of glycolipids, it is not surprising that she was a forerunner in MALDI-MS analysis of native and permethylated gangliosides. Both positive and negative ionization modes were explored for detection of intact molecular ions of the gangliosides [35]. Fragmentation of underivatized gangliosides was observed with MALDI, and the potential utility of the observed fragments for retrieving structural information was suggested. The choice of matrix compound and experimental conditions such as laser wavelength and power were reported to have a strong effect on the observed fragmentation, defining “hot” and “soft” MALDI matrices. Due to the requirement for both positive and negative ionization, the ganglioside studies led not only to identification of matrices suitable for both ionization modes among already known compounds but also to the discovery of alternative matrices such as 6-aza-2-thiothymine (ATT), 1,5- diaminonaphthalene (DAN), and 4-hydrazinobenzoic acid. Since then, these new matrix compounds have found many interesting applications. ATT was used for analysis of glycoconjugates, including acidic oligosaccharides and glycopeptides [36], sulfated oligosaccharides [37], derivatized oligosaccharides [38], glycoproteins [39], and sialylated gangliosides [40, 41]. ATT has also been reported to be a relatively soft matrix and useful to ionize non-covalent protein complexes [42, 43] and to study peptide-peptide interaction [44]. A recent study of the laser wavelength dependence showed that the optimal range for detection of peptide-peptide complexes was outside the main absorption band of ATT, between 220 and 250 nm and to smaller extent around 330 nm. While N_2_ lasers, emitting at 337 nm, can still be used, most modern MALDI TOF instruments use frequency-tripled Nd:YAG lasers emitting at 355 nm, thus operating outside of this range. Finally, DAN has recently been intensively used for in-source decay studies [45–49] and for disulfide linkage identification due to its ability of their partial in-plume reduction [50, 51].

ESI-MS [52, 53] quickly became a method of choice for the analysis of both native and derivatized glycoconjugates, especially for oligosaccharides, due to the gentle nature of the ionization mode. Upon collisional activation of the ESI molecular ions, both linkage and branching details could be obtained. Dr. Costello was among those who quickly realized the advantage of ESI over FAB-MS for oligosaccharide analysis and contributed with both transferring existing FAB experience and development of new methodologies for oligosaccharide analysis by ESI-MS [27, 54].

As new ESI- and MALDI-MS methods for analysis of released glycans emerged, there was a strong need to develop methods capable of glycan characterization at a specific glycosylation site. Dr. Costello used at an early stage MALDI-MS of HPLC-separated glycopeptides to characterize glycosylation sites on human α-1-acid glycoprotein [55]. This study showed for the first time, the power of MALDI-MS to determine the glycosylation patterns at individual glycosylation sites. MALDI-MS allowed for detection of not previously observed minor glycoforms for several of the glycosylated sites. (The detailed study of human α-1-acid glycoprotein comprised a well-characterized glycoprotein standard, which has since been used by many research groups for method development and validation.) A year later, the same approach was applied for the characterization of the glycosylation sites pattern of the (Na,K)-ATPase [56]. The analysis was extended to detection and identification of the sialylated glycopeptides. The power of MALDI-MS for the detection of glycosylation patterns and discovery of new glycoforms was shortly demonstrated by the analysis of human transferrin receptor, for which no previously known phosphorylated high-mannose structures were observed, but made clearly evident by the detection of molecular ions with a mass increase of 80 Da [57].

Well suited for analysis of mixtures and with its tolerance to salt and buffers, MALDI-MS was a good choice for glycoconjugate analysis. However, until introduction of post-source decay (PSD) in 1991 [58, 59], MALDI-TOF MS could only offer molecular mass determination without further structural information. Initially developed for peptide sequencing, the post-source decay (PSD) method was applied in its early days to the analysis of native oligosaccharides, showing the capabilities to provide both sequence and branching information [60, 61]. From her earlier studies, Dr. Costello knew that derivatization of glycoconjugates prior to MS not only results in improved detection sensitivity but also affects the fragmentation pattern, facilitating their structural characterization [62]. With analysis of permethylated oligosaccharides having just been established for ESI MS/MS by Dr. Costello’s group [27], she proceeded to develop PSD analysis for glycoconjugates. In contrast to ESI, which produces protonated ions from permethylated glycoconjugates, MALDI produces strongly cation-adducted molecular ions. Systematic studies of PSD analysis for sodiated ions of permethylated glycosphingolipids and oligosaccharides were carried out by Dr. Costello’s group [63, 64]. These studies showed that MALDI PSD MS/MS was a sensitive method capable of distinguishing different structural glycoforms, providing information on sequence, branching, and to a certain extent, on linkages.

ESI-MS of permethylated oligosaccharides remains as method of choice for sequencing of oligosaccharides, in part due to the most complete sequence information obtained from fragmentation of their protonated ions and in part to its ease of coupling with LC to facilitate analysis of complex mixtures. However, LC is not the only separation technique that is routinely used in analysis of glycoconjugates. Thin layer chromatography (TLC) is a well-established separation technique for glycolipid analysis and has been shown to be compatible with FAB-MS for separate species identification [65]. MALDI as a solid state ionization technique is very attractive for analysis of material from various surfaces, such as TLC plates and PVDF membranes. A method for MALDI TOF MS of TLC separated glycosphingolipids directly from TLC and after blotting to various membranes, was developed by Dr. Costello’s group [66]. MALDI TOF MS of the membrane-bound analytes showed improved sensitivity and offered structural analysis of detected analytes by PSD. Limited resolution and mass accuracy was observed due to surface irregularities, with the best results obtained for samples blotted to membranes. The low resolution and mass accuracy limits glycosphingolipid analysis, and thus the use of alternative mass analyzer, Fourier transform ion cyclotron resonance (FT ICR) MS, was thus employed. For MALDI FT ICR MS, the mass accuracy is independent of the initial energy spread for the analyte ions and resolution and mass accuracy are not degraded. However, to take advantage of high mass accuracy and resolution of FT MS, the problem of metastable decay of MALDI produced ions had to be solved. The same problem had been observed in orthogonal MALDI TOF instruments and was solved by the introduction of high-pressure MALDI ion sources. With MALDI FTICR MS, the delay between ion formation and detection is significantly longer compare to other instruments and can take up to several seconds. Thus, cooling of the MALDI ions is essential for analysis of glycoconjugates with FTICR MS. Dr. Costello’s group was the first to test high-pressure MALDI with FTICR MS. They designed and tested a high-pressure ion source that resulted in decreased metastable fragmentation [41]. Detection of intact molecular ion for multiply sialylated gangliosides was demonstrated. To facilitate direct TLC analysis by MS, the ion source capable to accommodate large targets was developed [67] and tested for the analysis of TLC-separated gangliosides. Dr. Costello’s interest and experience with various MALDI matrix compounds led to the testing of 20 different UV and 3 IR matrices in the study. Without elevated pressure, even the softest matrices resulted in metastable decomposition of sialylated GSL; in contrast, the elevated pressure MALDI allowed them to use even hot MALDI matrices with success.

Different ion activation modes are available for FTICR MS, dependent on instrument configuration. Sustained off-resonance irradiation collision-induced dissociation (SORI-CID) is one technique that is widely used. MALDI generates singly charged ions over a relatively broad *m*/*z* range compared to the multiply charged ions produced by ESI. This adds requirements for optimization for collisional activation analysis with SORI-CID over a broad frequency range. To facilitate structural analysis of MALDI produced molecular ions with SORI-CID, a simple calculation for the irradiation frequency offset was suggested [40].

Dr. Costello has always readily shared her knowledge on glycoconjugate analysis and been open to collaborations with other groups in development of new tools for their analysis [68, 69]. The studies by Dr. Costello’s and other groups clearly showed that both MALDI and ESI MS are well suited to detect the glycosylation profiles of various glycoconjugates. Both techniques offered sensitive detection and structural analysis, and it was natural to question whether the observed relative intensities correlate with glycoform abundances. Using permethylated glucose oligomers, Dr. Costello showed that relative abundances observed with ESI and MALDI correlated well with data obtained from fluorophore-assisted carbohydrate electrophoresis [54]. However, it was noted that the presence of negatively charged residues or substituents affected ionization and were discriminated in mixture analysis. Thus, compared to other ionization techniques, both MALDI and ESI were less prone to competitive ionization for glycoconjugates of similar structures, suggesting the feasibility of their quantitative measurements.

Quantification is a hot topic in mass spectrometry. It is generally regarded that MS does not allow direct quantification of compounds in mixtures. However, the findings published by Dr. Costello suggested that relative quantification of oligosaccharides can be feasible. A decade later, a multi-institutional study organized by HUPO Human Disease Glycomics/Proteome Initiative demonstrated that she was correct [70]. This multicenter study showed that relative quantification of permethylated oligosaccharides is possible by MS and give results comparable to traditional HPLC methods. Glycoform profiling at the glycopeptide level also showed good results, but was concluded still to be too complex an analysis not yet applicable outside of specialized laboratories. Thus, the continuous work and method development carried by laboratories specialized on glycoconjugate analysis are still crucial to the development of methods that can be applied widely within the MS community.

## Developments in Structural Mass Spectrometry

FTICR MS offers several useful fragmentation techniques, and Dr. Costello and co-workers have made substantial contributions as applied to the study of oligosaccharides and glycoconjugates. Earlier, Carr and Burlingame [71] had shown that low-energy collision activated dissociation (CAD; also called collision induced dissociation (CID)) of glycopeptides yielded abundant fragment ions derived from cleavages of glycosidic bonds, with little dissociation in the peptide backbone structure. In 1998, Zubarev et al. [72] introduced electron capture dissociation (ECD) and in 1999 further demonstrated ECD as an activation technique that generates fragment ions derived from peptide backbone with no loss of the glycan moiety [73]. Building on the CAD and ECD, the tandem MS methods were combined in a single high-resolution FTICR MS instrument by Swedish collaborators. Håkansson et al. in 2001 applied infrared multiphoton dissociation (IRMPD) and ECD to dissociation of N-glycopeptides derived from a glycosylated lectin [68].

**Figure 1:**
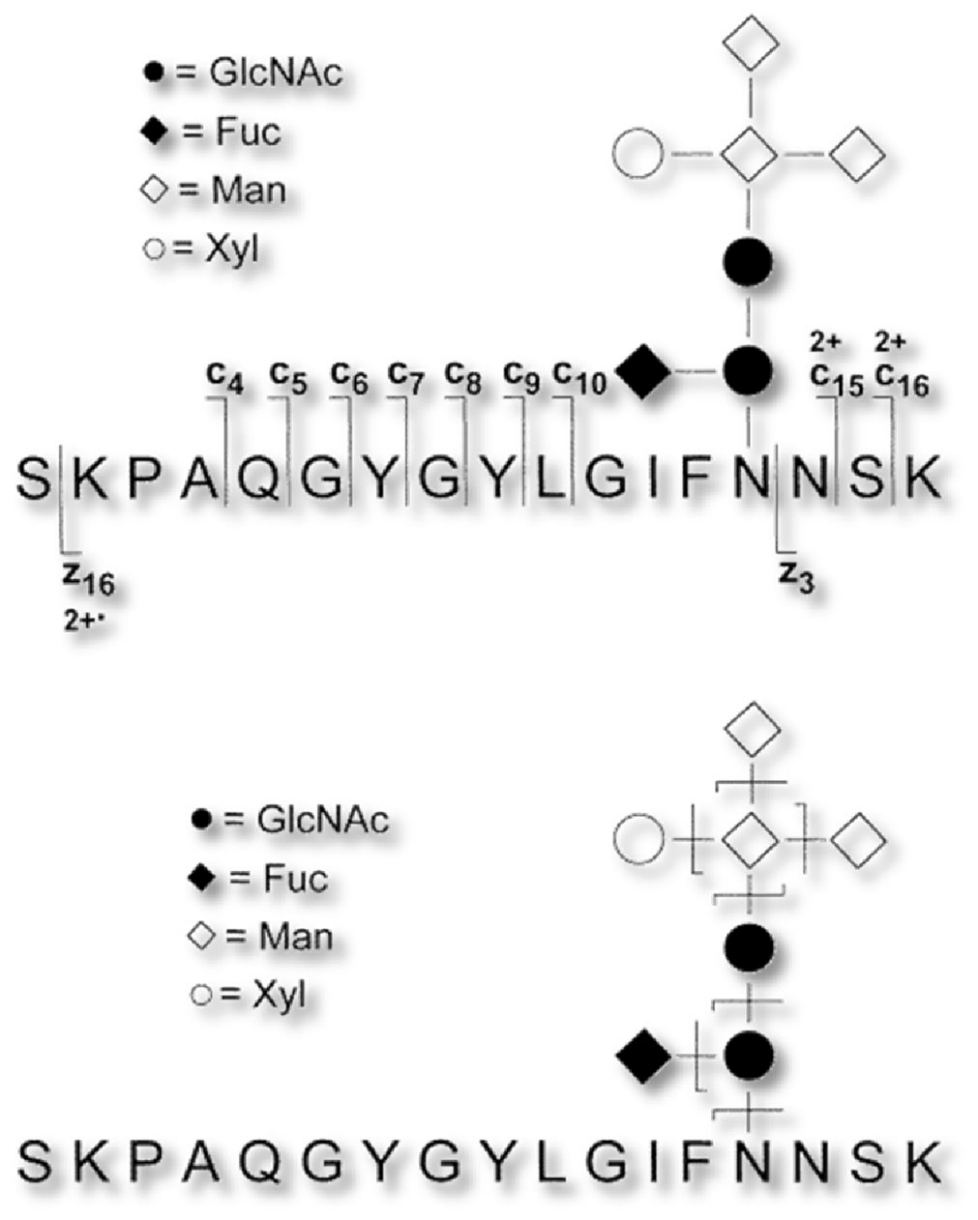
Fragmentation of an N-linked glycopeptide ECD (top) and IRMPD (bottom) demonstrate strict complementarity [68]

The advantage of IRMPD over CAD is that fast and efficient dissociation is obtained with no need to pulse gas into the ICR cell (which degrades the vacuum), permitting rapid fragmentation analysis. It was shown that IRMPD, similar to CAD, resulted mainly in cleavage of glycosidic linkages, thus revealing structural information about the glycan. Strictly, complementary behavior was observed for the fragmentation techniques IRMPD and ECD (Fig. 1). In addition to glycopeptide identification, the presence of three branch points in the glycan could be established based on the observed fragment ions, without enzymatic release of the oligosaccharide. The published ECD spectrum (Fig. 1) was the first example of backbone fragmentation of a peptide containing a multiply branched glycan without any observed cleavage of the glycosidic bonds.

Further developmental work of combined MS/MS approaches were presented in 2002 [74]. The data obtained demonstrated that ECD spectra of peptides containing more than 30% proline residues were simpler and thus easier to interpret compared to CID spectra. Proline-rich proteins are unusually difficult to sequence by mass spectrometry due to the high efficiency of cleavage at the amide bond on the N-terminal of proline residues and the consequently low relative abundance of fragments arising from cleavages at other amide bonds. Factors that limit the two methods of fragmentation include the complexity of information contained in the CID spectra and the low efficiency of ECD processes. A complementary approach using both decomposition methods provides more complete and interpretable sequence information and yielded > 93% sequence coverage for the 11-kDa proline-rich human salivary protein. In 2008, CAD and ECD were successfully applied by Dr. Costello’s group to characterize branched sodiated and permethylated *N*-linked glycans, such as high-mannose-type GlcNAc2Man5–9, and complex asialo- and disialylated-biantennary structures [75]. Dr. Costello’s impact in the development of complementary dissociation strategies for a full characterization of both glycopeptide and glycan have resulted in their widespread application today.

Finally, the resolution of exact isobars in the gas phase benefits greatly from ion mobility (IM) MS, and Dr. Costello has made important contributions to this modality in glycobiology. Separation of isomeric glycans by high-mobility resolution cross-sectional measurements FTICR MS [76], improved glycan isomer separations with metal ion adducts, and switched polarity IM-MS [77] are examples of technical advances. The development of a collisional cross-section (CCS) database for glycomics [78] initiated by Dr. Costello has simplified IM MS data analysis.

## A Framework for Glycan Nomenclature and Glycoinformatics

**Figure 2:**
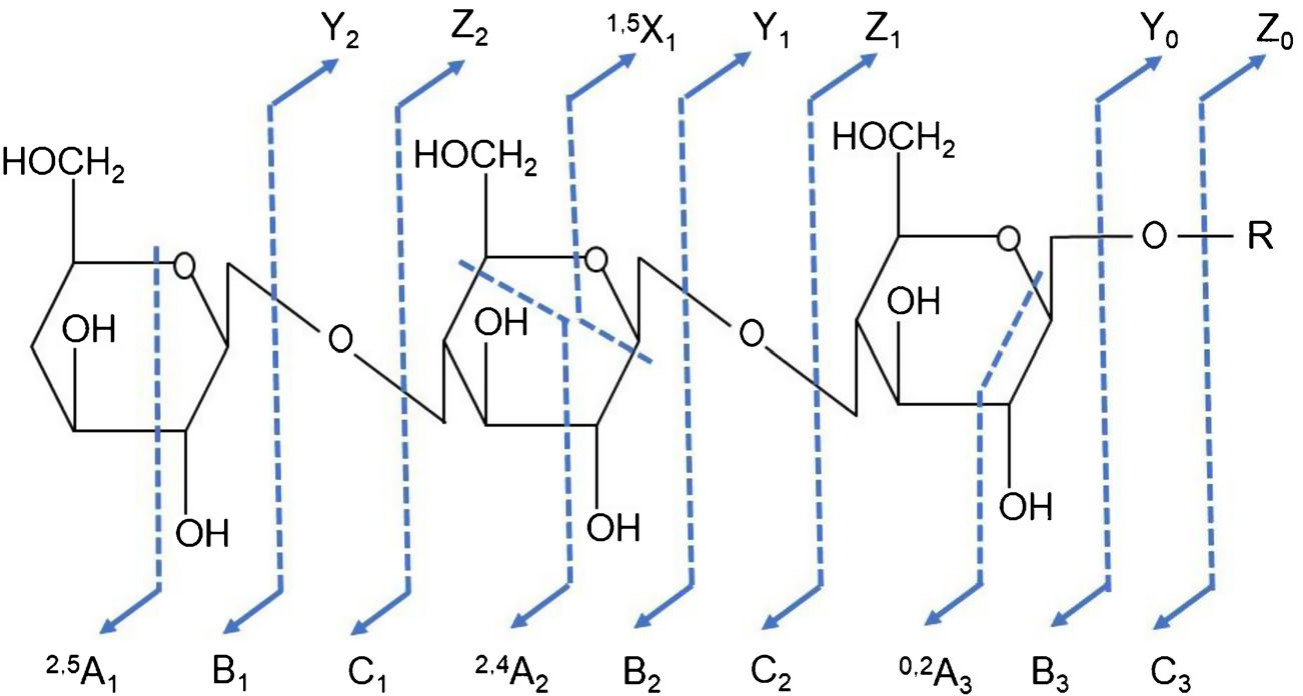
Oligosaccharide fragmentation nomenclature as developed by Domon and Costello [25]. The comprehensive system includes a code for dissociation between saccharide moieties as well as cross-ring fragmentation types

Every carbohydrate MS scientist knows the code for gas phase fragmentation of glycoconjugates in mass spectrometry. The introduction of a systematic nomenclature for fragmentation of carbohydrates and glycoconjugates by Domon and Costello in 1988 [26] is a perfect example of the fact that not all important scientific publications are published in mainstream bibliographic resources. This seminal publication about carbohydrate fragmentation is present neither in PubMed nor in Web of Science. Despite this, Google Scholar lists this publication as Professor Costello’s most highly cited paper (> 2000 citations; Fig. 2 below).

MS fragmentation of carbohydrates provides both glycosidic (B, C, Y, Z) and cross-ring (A, X) fragments to determine oligosaccharide sequences and linkage position. These structural features are unique for carbohydrate polymers as compared to other biological polymers. The fact that carbohydrates can be branched increases the complexity of the proposed nomenclature. However, its completeness made it applicable in 1988; few modifications have been necessary since its inception despite the introduction of newer fragmentation mechanisms for carbohydrates. This attests to Dr. Costello’s keen insight and ingenuity.

Emerging challenges for mass spectrometry are the organization of the large datasets, enabling communication by precise terminology, and implementation of data reporting standards to maintain research transparency. Public and private research foundations require open-access data; thus, an open-access glycodata repository is a prerequisite to further develop bioinformatic tools for the community. Dr. Costello’s involvement in the establishment of a Minimum Information Required for a Glycomic Experiment (MIRAGE) consortium (http://www.beilstein-institut.de/en/projects/mirage) [79] in 2011 is extended as a member of its advisory board. The consortium is sponsored and managed by the Beilstein Institute, with the aim to develop publication guidelines for interaction and structural glycomic data and glycomic data exchange formats. In addition to the MS guidelines [80], the consortium has also developed guidelines for sample preparation [81], glycan array data [82], and guidelines for glycomic HPLC and capillary electrophoresis. With the aim to get scientific journals to adopt these guidelines as part of paper submission procedures, the MIRAGE consortium was influential in developing a shorter version of the glycomic guidelines in 2012 and recruiting journals to follow the full MIRAGE guidelines to ensure the transparency of glycomic research. The MIRAGE guidelines for reporting have also been implemented in the glycomic spectrum library UniCarb-DB [83, 84] and GlyTouCan [85], the international glycan structure repository that requires a unique identifier for all identified reported glycans. Without Dr. Costello’s leadership, these developments would have taken much longer time to be realized.

At Boston University, Dr. Costello’s contributed to shared MS bioinformatic resources for glycomics and glycoproteomics. The resources are freely available online at http://www.bumc.bu.edu/msr/software/. Software tools for proteomic MS annotation and visualization [86], including post-translational modifications [87], are frequently utilized and referenced. Post-translational modifications, especially glycosylation, have been Dr. Costello’s highest-profile research. However, serving as the president for both the American Society for Mass Spectrometry (2002–2004) and the Human Proteome Organization (2011–2012), her contribution to other bioinformatic and database resources is illustrated by her participation in development of standards for MS reporting (mzXML) [88], open-access protein information [89, 90], and the field of immunopeptidomics [91]. Another important area of glycobiology is the structural elucidation of glycoaminoglycans by ESI-MS [92] and MS/MS [93], led by Joseph Zaia at the BUMC Mass Spectrometry Resource and launching his successful career.

Dr. Costello continues to contribute to the orthogonal use of ion mobility mass spectrometry to identify isobaric glycoisomers, increase precision in assignment of glycoconjugates sequences, and linkage position/configuration. This endeavor also includes the continuous development of the experimental CCS database (CCS) for carbohydrates and derivatives [78].

## Impact of Glycan Mass Spectrometry in Biology and Disease

The expansion of glyco-MS and enabling technologies have had a broad impact on the molecular characterization of biological isolates from human pathogens and human samples, providing specific molecular epitopes for further study. In this area, Dr. Costello has had important contributions to the characterization of novel phosphosphingolipids [94] and lipophosphoglycan-like glycoconjugates of *Trichomonas* species [95], the *Giardia* cyst wall protein 1-a lectin which binds a GalNAc homopolymer [96], the discovery of *O*-fucosylated glycoproteins near the nuclear pore complexes of *Toxoplasma gondii* [97], *N*-linked glycans in *Cryptosporidium parvum* unique structures as vaccine targets [98], the *N*-linked glycome of *Caenorhabditis elegans* [99], and the successful characterization of *Neisseria meningitidis* pilins by top-down mass spectrometry [100]. One of her most recent advances in human biology is the discovery of the carbohydrate-dependent mode of B cell receptor signaling [101].

## Summary

Dr. Costello has been a leading figure in the field of biological mass spectrometry since she entered the scientific arena decades ago. She has contributed to the development of analytical technologies related to biomolecules, especially glycoconjugates, including derivatization strategies for labile biomolecules, use of soft ionization techniques, nomenclature for glycoconjugate MS/MS fragment ion data, and engagement in bioinformatic approaches to glyco-MS data analysis. She has mentored many students from around the globe. Her impact has been great and continues to grow, making biological mass spectrometry an essential tool in glycoconjugate structural studies, especially microbiology and human biology. Her work to continuously stretch the boundaries of what mass spectrometry can do for life science with the aid of bioinformatics and databases is an inspiration for all of us. Her contribution in laying out the foundation for these resources impacts MS today and promises to have a lasting effect on the future of biological mass spectrometry.
